# Cleavage of HMGB1 by Proteolytic Enzymes Associated with Inflammatory Conditions

**DOI:** 10.3389/fimmu.2020.448262

**Published:** 2020-12-16

**Authors:** Agnieszka Sowinska, Merlin Rensing, Lena Klevenvall, Manoj Neog, Peter Lundbäck, Helena Erlandsson Harris

**Affiliations:** Division for Rheumatology, Center for Molecular Medicine, Department of Medicine Karolinska Institutet, Karolinska University Hospital in Solna, Stockholm, Sweden

**Keywords:** high mobility group box 1, neutrophil elastase, cathepsin G, matrix metalloproteinase-3, juvenile idiopathic arthritis, proteolytic cleavage

## Abstract

Extracellular HMGB1 acts as an alarmin in multiple autoimmune diseases. While its release and functions have been extensively studied, there is a substantial lack of knowledge regarding HMGB1 regulation at the site of inflammation. Herein we show that enzymes present in arthritis-affected joints process HMGB1 into smaller peptides *in vitro*. Gel electrophoresis, Western blotting and mass spectrometry analyses indicate cleavage sites for human neutrophil elastase, cathepsin G, and matrix metalloproteinase 3 within the HMGB1 structure. While human neutrophil elastase and matrix metalloproteinase 3 might alter the affinity of HMGB1 to its receptors by cleaving the acidic C-terminal tail, cathepsin G rapidly and completely degraded the alarmin. Contrary to a previous report we demonstrate that HMGB1 is not a substrate for dipeptidyl peptidase IV. We also provide novel information regarding the presence of these proteases in synovial fluid of juvenile idiopathic arthritis patients. Correlation analysis of protease levels and HMGB1 levels in synovial fluid samples did not, however, reveal any direct relationship between the recorded levels. This study provides knowledge of proteolytic processing of HMGB1 relevant for the regulation of HMGB1 during inflammatory disease.

## Introduction

High Mobility Group Box 1 protein (HMGB1) is a prototypical alarmin which is secreted by activated immune cells and passively released by damaged cells. Its extracellular inflammatory properties have been studied in multiple inflammatory diseases (1, 2). Intra-articular administration of recombinant HMGB1 into the knee joints of mice induces destructive arthritis, while administration of monoclonal anti-HMGB1 antibody or the antagonistic box A peptide can ameliorate inflammatory symptoms (3–6). Administration of HMGB1-neutralizing agents also provides significant protection in animal models of experimental sepsis, drug-induced liver injury, ischemia reperfusion injury and trauma (1, 7). HMGB1 contains conserved cysteine residues at position 23, 45, and 106, and the redox state of these cysteines determine how HMGB1 functions as a proinflammatory mediator (8).

HMGB1 has a highly conserved sequence consisting of three distinct structures: the DNA binding domains box A and box B, as well as an acidic tail at the C-terminal that is comprised of glutamic and aspartic acid residues. Dynamic interaction of the C-tail with amino acid residues within box A, box B, and within the linker region between box B and the C-tail affects both the stability of the protein and the DNA binding properties of the A and B boxes. HMGB1 in which the C-tail interacts with box A and box B has a more stable structure as compared to the “unbound” form (9–11).

**Figure 1:**
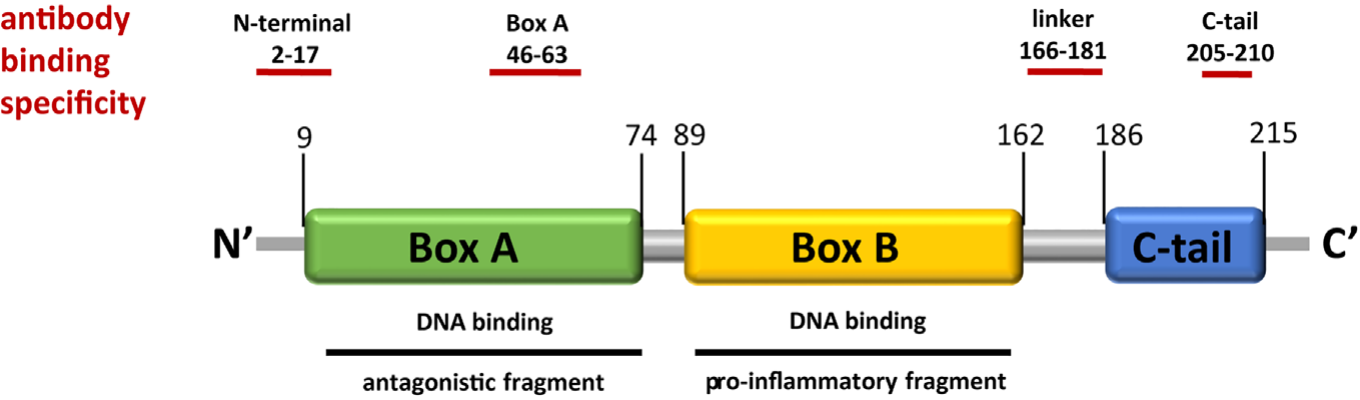
HMGB1 structure indicating functional domains and antibody epitopes. HMGB1 is a 25-kDa protein with a helical structure and consists of two DNA binding domains, box A and box B, as well as an acidic C-tail which can interfere with DNA binding. Different regions of the protein are ligands to immune receptors. While box B is known to have cytokine-like activity, free box A has been shown to act as an antagonist. Several antibodies have been developed to target different epitopes of HMGB1, those used in this study are indicated above.

Not only is full-length HMGB1 biologically active with potentially harmful, inflammatory effects but its peptides can also have inflammatory features. Studies with recombinant peptides show that box B harbors the cytokine-inducing property through its interaction with MD2/TLR4 (12), while box A on the other hand has the ability to inhibit proinflammatory activity of the full-length protein (13, 14) (**Figure 1**). Earlier reports of the anti-bacterial properties of HMGB1 attributed this feature to the acidic C-terminal tail (15). Full-length HMGB1, as well as box A and box B, can form complexes with other molecules, for example LPS and IL-1β, and thereby augment their inflammatory features (16). However, the existence of functional HMGB1 peptides *in vivo* is not as yet proven.

While HMGB1 release and its functions in inflammatory diseases have been extensively studied, less is known regarding how extracellular HMGB1 activity is regulated during disease. A recent study reports that haptoglobin, an acute phase extracellular hemoglobin-binding protein, binds circulating HMGB1 and thereby protects against sepsis (17). Thrombomodulin was earlier demonstrated to inhibit the inflammatory features of HMGB1 through interaction of its lectin-like domain with HMGB1 (18–20).

To date there are no studies focusing on the downregulation of HMGB1 at the focal site of inflammation. We hypothesized that proteolytic cleavage of HMGB1 could play important functions in the regulation of HMGB1 activity. Firstly, protein degradation would promote clearance of HMGB1 from the site of inflammation. Secondly, partial degradation could also result in certain receptor binding sites being more accessible and thereby improve the binding of HMGB1 to its receptors (21). Thirdly, it is also possible that enzymatic processing of HMGB1 could result in antagonistic HMGB1-derived peptides being formed with similar functions as “free” box A (22).

We set out to investigate proteolytic regulation of HMGB1 by proteases associated with chronic inflammatory disease, using JIA as a model disease. Four proteases associated with arthritis, neutrophil-derived human elastase (HNE) and Cathepsin G (CG), fibroblast-derived matrix metalloproteinase 3 (MMP-3) and the serine protease dipeptidyl peptidase-IV (DPP-IV), were investigated for their ability to process HMGB1. We characterized the resulting peptide patterns and correlated levels of HMGB1 with HNE, CG, MMP3 and DPP-IV levels in JIA joints.

## Materials and Methods

### Prediction of Protease Cleavage Sites in HMGB1 and Selection of Proteases Investigated

For an initial prediction of proteases with the potential to cleave HMGB1 we used the online software Protease specificity prediction server PROSPER (PROSPER, Monash University; https://prosper.erc.monash.edu.au/). PROSPER performs *in* *silico* prediction of protease substrates and cleavage sites predictions covering 24 proteases and the four major protease families (23)). The sequence of HMGB1 (UniProtKB-P09429, HMGB1_HUMAN) was uploaded to the online server. Based on PROSPER-identified proteases together with literature studies regarding proteases associated with inflammatory conditions in general and in arthritis in particular, we selected the proteases HNE, CG, MMP3 and DPP-IV for further investigation.

### Recombinant Protein Production

Human HMGB1 cDNA was cloned into the pETM-11 vector and expressed in *Escherichia coli* strain BL21 (DE3) cells. The protein expressed a 6-residue N-terminal histidine tag with a tobacco etch virus (TEV) cleavable linker and was purified by FPLC using Ni-sepharose affinity chromatography (HisTrap HP, GE Healthcare, Uppsala, Sweden) in an ÄKTA explorer (GE Healthcare). The histidine tag was cleaved using TEV protease (Sigma-Aldrich, Stockholm, Sweden) at a ratio of 1:20. Proteolytic TEV cleavage leaves a GA scar at the N-terminal. Endotoxins were removed using Triton-X114 two phase extraction. Protein purity was confirmed using SDS-PAGE gel electrophoresis analysis (21).

### Enzymatic Reactions

CG (Cat: 219373), HNE (Cat: 324681) and MMP3 (catalytic domain, Cat: 444217) were obtained from Merck (Millipore, Billerica, MA, USA). DPP-IV (Cat: D4943) was obtained from Sigma (Sigma-Aldrich) or from BioVision (Cat: 4709, AH Diagnostics, Stockholm, Sweden). Enzyme reconstitution and reaction buffers were prepared according to the supplier’s recommendations. CG and recombinant HMGB1 were mixed at a molar ratio of 1:80 in phosphate-buffered saline (PBS), pH 7.4 and the reaction was carried out at 37°C. HNE and recombinant HMGB1 were mixed at a molar ratio of 1:50 in 100 mM Tris-HCl, 500 mM NaCl, pH 7.5 and the reaction was carried out at 25°C. MMP3 and recombinant HMGB1 were mixed at a molar ratio of 1:22 in 10 mM CaCl_2_, 1 μM ZnCl_2_, pH 7.5 and the reaction was carried out at 37°C. DPP-IV and recombinant HMGB1 were mixed in 50 mM Tris-HCl, 1 mM EDTA, pH 7.4 at a molar ratio of 1:28 and the reaction was carried out at 37°C. Reactions were performed in sterile 1.5-ml tubes and heat block was used in order to maintain constant temperature. All enzymatic digestions were performed with a set amount of 2 µg HMGB1. The reactions were stopped by addition of 5 µl of Laemmli sample buffer containing β-mercaptoethanol to 10 μl sample and heat inactivation at 95°C for 10 min.

Activity of DPP-IV was verified in an activity assay according to the manufacturer’s instructions (Promega DPP-IV Glo assay, G8350, Promega Biotech AB, Nacka, Sweden).

### SDS-PAGE Gel Electrophoresis

Digested samples were analyzed by SDS-page gel electrophoresis using 4% to 20% gradient Tris-Glycine gels (Bio-Rad Laboratories Inc, CA, US). Gels were stained with Coomassie blue and analyzed using Image Lab 6.0.0 (Bio-Rad Laboratories Inc).

### Western Blotting

SDS-PAGE gels were loaded with sample volumes corresponding to 200 to 500 ng digested HMGB1 and run at 250 V for 25 min and transferred to nitrocellulose blotting membranes (GE Healthcare) at 100 V for 1 h. Membranes were subsequently blocked overnight in 5% dry milk in TBS-T, incubated with selected primary antibodies (1 μg/ml) for 1 h at RT followed by incubation with secondary antibodies coupled to HRP (1:10000 dilutions) for 1 h at room temperature. Chemiluminescence was detected by ChemiDoc MP imaging system (Bio-Rad Laboratories Inc). The following primary antibodies were used: Ab67281 (rabbit polyclonal against HMGB1 amino acids 2 to 17, Abcam, Cambridge, UK), ab 2G7 (mouse monoclonal IgG2b, against HMGB1 amino acids 46 to 63 (24)), Ab K25 (rabbit polyclonal against HMGB1 amino acids 161–188, in house production) and ab #10–22 (rat monoclonal IgG2a against HMGB1 amino acids 205–210, a kind gift from Prof. Nishibori, Okayama University, Japan (25)), (**Figure 1**). The following secondary antibodies were used: polyclonal donkey anti rabbit IgG-HRP (711-035-152, Jackson Immunoresearch Laboratories Inc), polyclonal rabbit anti-mouse IgG-HRP (P0260, Dako Cytomation), polyclonal rabbit anti rat Ig-HRP (P0163, Dako Cytomation).

### Mass Spectrometry Analysis

Protein digestion mixtures were separated by SDS-PAGE (4 μg protein/time point) and specific bands were excised. All bands were excised at the 60-min time point, except fragment I/MMP3 cleavage which was excised at the 30-min time point. In-gel trypsin digestion was performed and thereafter the samples were resolved in 30 µl 0.1% formic acid prior to nanoLC-MS/MS. The resulting peptides were separated on a C18-column and electrosprayed online to a QEx-Orbitrap mass spectrometer (Thermo Finnigan) with 35 min gradient. Tandem mass spectrometry was performed applying higher-energy collisional dissociation (HCD) fragmentation.

MS/MS data were matched to a sequence database (*Homo sapiens* proteome extracted from Uniprot, release December 2017) using the Sequest algorithm, embedded in Proteome Discoverer 1.4 (Thermo Fisher Scientific). This sequence identifies HMGB1 as a 215 amino acid long protein with a methionine in position 1. The search criteria for protein identification were set to at least two matching peptides of 95% confidence level per protein. Compared to standard search settings, peptides down to four amino acids length were accepted. The excised gel bands were delivered to SciLife Lab, MS facility, Uppsala, Sweden, where the digestion, MS analysis and protein identification was performed.

### 3D Modeling of Predicted Cleavage Sites

HMGB1 and its cleavage sites was visualized in 3D using The PyMOL Molecular Graphics System, Version 1.8 Schrödinger, LLC. A solution NMR structure of HMGB1 corresponding to amino acids 1 to 166 (i.e. lacking the C-tail part of the molecule) was used as a model (2YRQ accession in Protein Data Bank, https://www.rcsb.org/).

### Synovial Fluid Samples

Synovial fluid (SF) from 16 juvenile idiopathic arthritis (JIA) patients with active disease was collected at Astrid Lindgren’s Children Hospital, Stockholm, Sweden as part of the sample collection JABBA. Median patient age was 11 years (range: 3–18). Mean disease duration was 30.11 months (range: 0–180). Patients were enrolled according to ILAR criteria, and JIA subtype distributions were as follows: oligoarthritis (75%), polyarthritis (12.5%), and undifferentiated arthritis (12.5%). SF was collected in citrate tubes, filtered and centrifuged to obtain cell-free SF and stored at −80°C until use.

Informed consent was given by both parents and children to participate in the study. The study is in accordance with the Helsinki declaration and was approved by the North Stockholm Ethical Committee in Stockholm, Sweden (Dnrs 2009-1139-31-4 and 2010-165-31-2.)

### HMGB1 and Enzyme Detection in Synovial Fluid (SF) of JIA

HMGB1 levels in JIA SF samples were determined using a commercial ELISA according to the manufacturer’s instructions (Shino-test, IBL International, Hamburg, Germany). Levels of HNE, MMP3 (duosets # DY9167-05 and # DY513, R&D Systems, Minneapolis, MN, USA), CG (NBP2-60614, Novus Biologicals, Bio-Techne, Stockholm, Sweden) and DPP-IV (RAB 0147, Sigma Aldrich) were measured in SF using ELISA systems according to the manufacturer’s instructions.

### Statistical Methods

Due to non-normality of the data Spearman correlation test of recorded levels of HMGB1 and enzymes in SF specimens was performed using GraphPad Prism 6.0.

## Results

### Proteases Predicted to Cleave HMGB1

Analysis of the HMGB1 sequence using the protease specificity prediction server PROSPER revealed a number of proteases with the potential of cleaving HMGB1. 5 serine proteases, 2 cysteine proteases and 3 matrix metalloproteinases were predicted (**Table 1**). HNE, CG and MMP3 were selected for further investigation together with DPP-IV. DPP-IV has previously been reported to cleave HMGB1 (26) but was not predicted by PROSPER.

**Table 1:** Proteases predicted to cleave HMGB1.

Merops ID	Protease name	Position	P4-P4′ site	N-fragment (kDa)	C-fragment (kDa)
C01.036	CK	39	VNSF/EFSK	4.78	21.35
C01.036	CK	14	GKMS/SYAF	1.84	24.29
C02.001	Calpain 1	52	WKTM/SAKE	6.42	19.71
M10.003	MMP2	101	PPSA/FFLF	12.25	13.87
M10.004	MMP9	11	KPRG/KMSS	1.49	24.63
M10.004	MMP9	150	KAAK/LKEK	18.25	7.87
M10.004	MMP9	128	VAKK/LGEM	15.65	10.48
M10.004	MMP9	145	QPYE/KKAA	17.73	8.40
M10.004	MMP9	103	SAFF/LFCS	12.55	13.58
M10.004	MMP9	101	PPSA/FFLF	12.25	13.87
M10.004	MMP9	20	AFFV/QTCR	2.56	23.57
M10.005	MMP3	161	DIAA/YRAK	19.54	6.58
M10.005	MMP3	34	HPDA/SVNF	4.24	21.88
M10.005	MMP3	120	HPGL/SIGD	14.74	11.39
S01.001	Chymotrypsin A	16	MSSY/AFFV	2.09	24.04
S01.001	Chymotrypsin A	78	MKTY/IPPK	9.63	16.50
S01.131	HNE	20	AFFV/QTCR	2.56	23.57
S01.133	CG	38	SVNF/SESF	4.69	21.44
S01.133	CG	120	HPGL/SIGD	14.74	11.39
S26.008	CG	75	EREM/KTYI	9.23	16.89
S26.008	GP 1	40	NFSE/FSKK	4.91	21.22
S26.008	TPP	171	PDAA/KKGV	20.72	5.41
S26.008	TPP	207	EDED/EEED	25.15	3.60
S26.008	TPP	35	PDAS/VNFS	4.33	21.80
S26.008	TPP	160	KDIA/AYRA	19.47	6.65
S26.008	TPP	69	ADKA/RYER	8.37	17.76
S26.008	TPP	198	EDEE/EEED	25.41	3.60
S26.008	TPP	64	EDMA/KADK	7.86	18.27

Proteases predicted to cleave HMGB1 using the protease specificity prediction server PROSPER. Merops ID defines the protease identity and classification in the MEROPS peptidase database (www.ebi.ac.uk/merops). CK; Cathepsin K, MMP; matrix metalloproteinase, HNE; human elastase 2; CG; Cathepsin G, GP 1; glutamyl peptidase 1, TPP; thylakoid processing peptidase.

### Elastase (HNE) Cleaves HMGB1 at Multiple Sites

**Figure 2:**
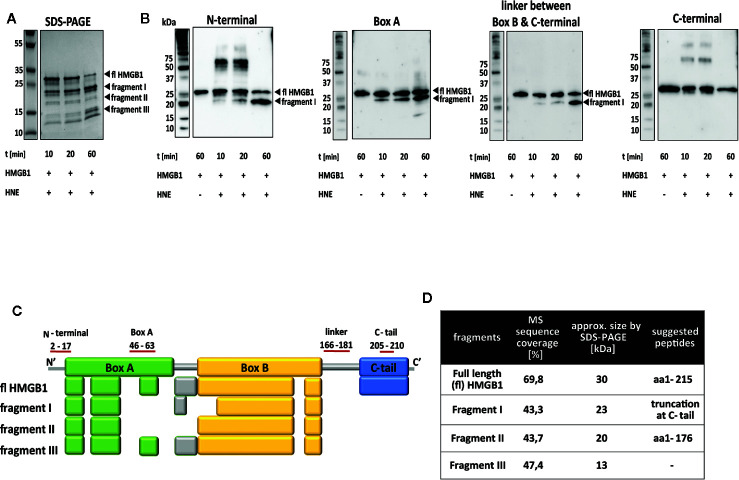
HNE cleaves HMGB1 at the C-terminal part and within box A. **(A)** Left panel: SDS-PAGE showing full length HMGB1. Right panel: SDS-PAGE showing the rapid cleavage by HNE over time. A larger fragment (I) appeared early and increased in strength during the studied time frame. A smaller fragment (III) also increased in strength during the studied time frame while an intermediate-sized fragment (II) appeared equal in strength throughout the cleavage reaction. **(B)** Western blotting demonstrating the presence of different protein regions in the HNE-generated HMGB1 fragments. Fragment I contained both the N-terminal and the box A epitopes but not the C-terminal tail epitope. A lower Mw fragment with an apparent size of 15 kDa was detected in Western blotting with the antibody against Box A but with none of the other antibodies used. **(C)** Gel bands of HMGB1 fragments I to III were analyzed by mass spectrometry and the resulting peptides were compared to peptides detected in the full length protein. Colored boxes refer to the functional domains of HMGB1 in which peptides could be identified (Box A: Green, linker region: grey, box B: yellow, C-terminal tail: blue). **(D)** Suggested cleavage sites based on data in **(A–C)** together with literature and database searches.

Processing of HMGB1 with HNE resulted in a distinct peptide pattern. During the first minutes of the reaction, a larger fragment with an apparent molecular weight (Mw) of 23 kDa (fragment I, **Figure 2A**) dominated while after 60 min incubation a lower Mw fragment of approximately 13 kDa appeared as the final product of the reaction (fragment III, **Figure 2A**). A constant intermediate product was also detected (fragment II, **Figure 2A**). Analysis of the fragments using antibodies against different HMGB1 epitopes demonstrated a fragment containing the N-terminal region, the box A region and the linker region between box B and the C-tail (**Figure 2B**). The Mw of the peptide corresponded to the major cleavage product evident in SDS-PAGE (**Figure 2A**, fragment I). A complete lack of signal for the C-terminal region at all time points indicates that C-terminal tail truncation is an early event.

In order to investigate the possible cleavage sites, full length HMGB1 and the three fragments were analyzed by mass spectrometry. Analysis of full length HMGB1 created a mapping reference for the fragments with a sequence coverage of 69.8%. It also demonstrated that the N-terminal region could never be detected since cleavage with trypsin, the enzyme used in the mass spectrometry protocol, did not generate any peptide fragments in this region (**Figure 2C**). The lack of C-terminal peptides in fragments I-III confirmed that HNE-mediated C-terminal truncation was an early event (**Figures 2A, B**, fragment I, sequence coverage 43.2%). Multiple peptides spanning over box A and box B were detected in fragments II and III. Sequence coverage was 43.7% and 47.4%, respectively. Comparison of detected peptides with the fragment sizes estimated by SDS-PAGE suggested that there were more than one HMGB1 peptide of similar size in these fragments. The poor resolution of SDS-PAGE makes it challenging to resolve these fragments for separate extraction/isolation (**Figure 2C**, fragments II and III). Cleavage site prediction for HNE and HMGB1 by *PROSPER* indicated one potential cleavage site, at V20 (**Table 1**). This was not evident in our MS analysis. However, the lack of signal for the N-terminal antibody in Western blotting might preclude detection of cleavage in position V20. Earlier published reports suggest HNE cleavage sites at A34/A94/A101/G130/L120/V175/V176 in position P1 of substrates (27–30). Thus, based on Western blotting, mass spectrometry data and literature searches, we suggest that HNE prefers the cleavage site V175 or V176 at position P1 (**Figure 2D**). Cleavage at position V176 would result in a fragment with intact N-terminal of a predicted size of 19.7 kDa. Fragment II could consist of peptides cleaved either at V20 or A34 resulting in peptides with Mws of 17.4 kDa and 15.7 kDa. Fragment III could similarly as fragment II consist of peptides cleaved either at V20 or A34 and potentially at the cleavage sites suggested in literature previously. The calculated fragment sizes agree with the apparent fragment sizes approximated from Western blotting.

A lower Mw fragment with an apparent size of 15 kDa was detected in Western blotting with the antibody against Box A but with none of the other antibodies used. It was faintly visible at 60 min but not at the earlier time points (**Figure 2B**). As MS analysis of fragment II indicated overlaying peptides, potentially cleaved at either V20 or A34 within Box A, it is possible that either of the cleavage sites was more pronounced at later time points resulting in a peptide faintly visible only at the 60-min time point.

### Cathepsin G (CG) Rapidly Degrades HMGB1

**Figure 3:**
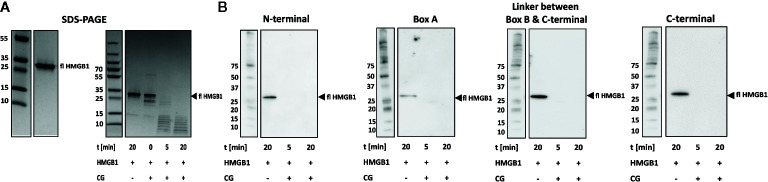
CG rapidly degrades HMGB1. **(A)** Left panel: SDS-PAGE showing full length HMGB1. Right panel: SDS-PAGE showing the rapid degradation of HMGB1 by CG over time. **(B)** Western blotting with antibodies recognizing epitopes within the N-terminal, the Box A, the linker between Box B and C-terminal and within the C-terminal regions resulted in detection of fragments.

The use of PROSPER indicated that the serine protease CG can cleave HMGB1 at three sites, F38, M75, and L120 in P1 positions. We could verify cleavage with CG *in vitro* by SDS-PAGE. However, at a molar ratio as low as 1:80, CG completely degraded HMGB1 within 5 min (**Figure 3A**). This observation was confirmed by Western blotting as no positive signal was evident with any of the epitope-specific antibodies used (**Figure 3B**). Due to the fast kinetics of the reaction and poor resolution of SDS-PAGE, we decided not to investigate the cleavage sites by mass spectrometry. It is noteworthy that a band of approximately 25 kDa occurs at t = 0, ie immediately after the addition of CG, similar in size to fragment 1 generated by HNE cleavage.

### Matrix Metalloproteinase 3 (MMP3) Cleaves HMGB1 at Multiple Sites

**Figure 4:**
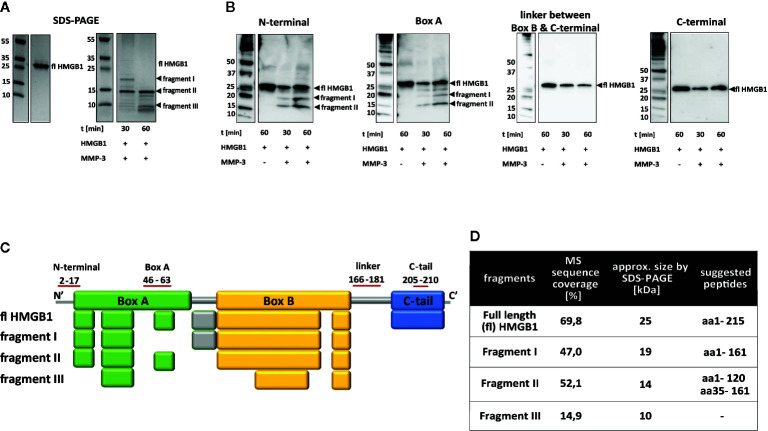
MMP3 cleaves HMGB1 at multiple sites. **(A)** Left panel: SDS-PAGE showing full length HMGB1. Right panel: SDS-PAGE showing cleavage by MMP3 over time. Two distinct fragments of approximately 19 and 14 kDa (I and II) appeared at time point 30 min. At 60 min, fragment I had disappeared while fragment II was still the dominant fragment. **(B)** Western blotting of HMGB1 fragments after processing with MMP3. A fragment corresponding to fragment I in size was detected by the antibodies specific for epitopes in the N-terminal region and in the box A region. In agreement with the SDS-PAGE kinetic pattern, the staining was stronger at the 30-min time point than the 60-min time point. Antibodies specific for epitopes within the B-to-C linker region and the C-terminal region only detected full length HMGB1 and none of the fragments. **(C)** Gel bands of HMGB1 fragments I-III were analyzed by mass spectrometry and the resulting peptides were compared to peptides detected in the full length protein. Colored boxes refer to the functional domains of the HMGB1 in which peptides could be identified (Box A: Green, linker region: grey, box B: yellow, C-terminal tail: blue). **(D)** Suggested cleavage sites based on data in **(A–C)** together with literature and database searches.

Exposure of HMGB1 to MMP3 processing resulted in the rapid formation of two fragments of approximately 19 and 14 kDa in Mws, visible after 30 min. Several smaller fragments were also visible both after 30 min and after 60 min (**Figure 4A**, fragments I, II, III and other fragments). However, the major reaction product was the 14-kDa fragment (fragment II), which was also evident after 60 min when the 19-kDa fragment (fragment I) had disappeared (**Figure 4A**). Western blotting analysis revealed a fragment of approximately 14 kDa being recognized by both the N-terminal specific antibody and the box A-specific antibody, corresponding to fragment II. Additionally, a fragment of approximately 19 kDa was detected after 30- and 60-min digestion with the N-terminal- and box A-specific antibodies, corresponding to fragment I (**Figure 4B**). None of the fragments I or II could be detected in Western blotting with the antibodies specific for the B-to-C linker region and the C-terminal tail region, respectively. This indicates a rapid cleavage within the B-to-C linker region resulting in C-terminally truncated HMGB1. Fragment III (**Figure 4A**) was undetectable by blotting.

Mass spectrometry analysis of fragments I and II confirmed the Western blot results that these fragments do not contain the box B-to-C linker region resulting in a C-terminally truncated HMGB1. Peptide coverage by MS analysis (47%) and fragment size estimated for fragment I by SDS-PAGE supported the conclusion that fragment I contained the N-terminal region, the box A region and the box B region. For fragment II the MS peptide coverage (52.1%) indicated a similar peptide as fragment I. However, this is not in total agreement with the fragment size estimated by SDS-PAGE, which is slightly lower with an approximate Mw of 14 kDa. This might indicate an additional cleavage site. Western blotting data suggests this cleavage site to be within box B. Mass spectrometry analysis of fragment III (peptide coverage 14.9%), indicated an overlay of different smaller peptides within the analyzed fragment, as peptides detected by MS did not correspond to the fragment size estimated by SDS-PAGE (**Figures 4A, C**).

Cleavage prediction using PROSPER indicated three cleavage sites for MMP3; A161, A34, and L120 in position P1. Based on Western blotting, mass spectrometry data, PROSPER predictions and literature searches, we suggest that MMP3 has multiple cleavage sites within HMGB1. The most preferred cleavage site removes the C-terminal tail by cutting at A161 and creates a 19-kDa fragment detected by N-terminal and box A-specific antibodies (fragment I, **Figure 4**). Thus, both HNE and MMP3 have a cleavage preference which results in rapid removal of the C-terminal tail. The smaller fragment recognized by the same antibodies as described above, fragment II, can be a result of cleavage at L120 in position P1 (**Figure 4D**). We cannot exclude the possibility of additional cleavage sites for MMP3 within the HMGB1 structure. If HMGB1 is cleaved at both A34 and A161 in P1 positions then the resulting peptide would be of similar size to the peptide corresponding to amino acids M1-L120, but this was only seen by Western blotting with the box A specific antibody. Moreover, cleavage at E61 would create a peptide spanning from D62 to A161, approximately 10 kDa in size, which would not be recognized by any of the antibodies.

Fragment III contained both box A peptides and box B peptides, indicating that the fragment contained multiple smaller peptides less than 10 kDa in size.

### DPP-IV Does Not Cleave HMGB1

**Figure 5:**
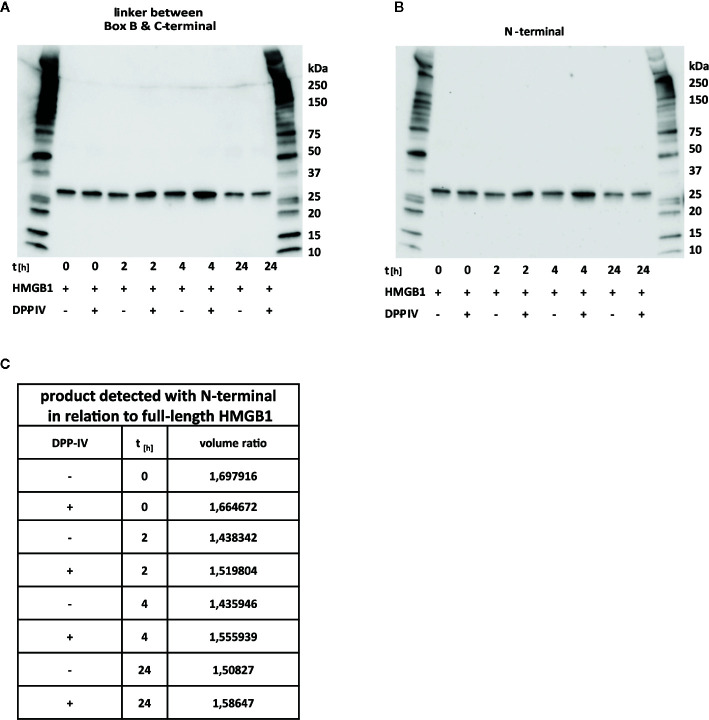
DPP-IV does not cleave HMGB1. HMGB1 was incubated with DPP-IV up to 24 h without any detectable sign of processing occurring. **(A)** Western blotting with an antibody recognizing the B-to-C linker region. **(B)** Western blotting with an antibody recognizing the N-terminal epitope aa 2–17. **(C)** Calculated signal ratios for Western blotting results in **(A, B)** reveal equal signals from reactions with DPP-IV and control reactions.

To verify a previous report that HMGB1 is cleaved by DPP-IV and to define the fragments generated, we reproduced the *in vitro* system utilized in the report (26). We could not record any cleavage occurring despite incubation times up to 24 h (**Figures 5A, B**). The activity of the enzyme was verified in an activity assay (**Supplemental Figure 1**). To further verify whether cleavage close to the N-terminal region occurred, as stated in the previous report, we performed Western blotting both with the polyclonal antibody binding to the B-to-C linker region (**Figure 5B**) and with the antibody specific for the N-terminal (**Figure 5C**) and calculated signal ratios obtained by image analysis (**Figure 5C**). The ratio was constant over time, thus further supporting the notion that DPP-IV does not cleave HMGB1.

### HMGB1 Is Detected in Synovial Fluid from Juvenile Idiopathic Arthritis Patients

**Figure 6:**
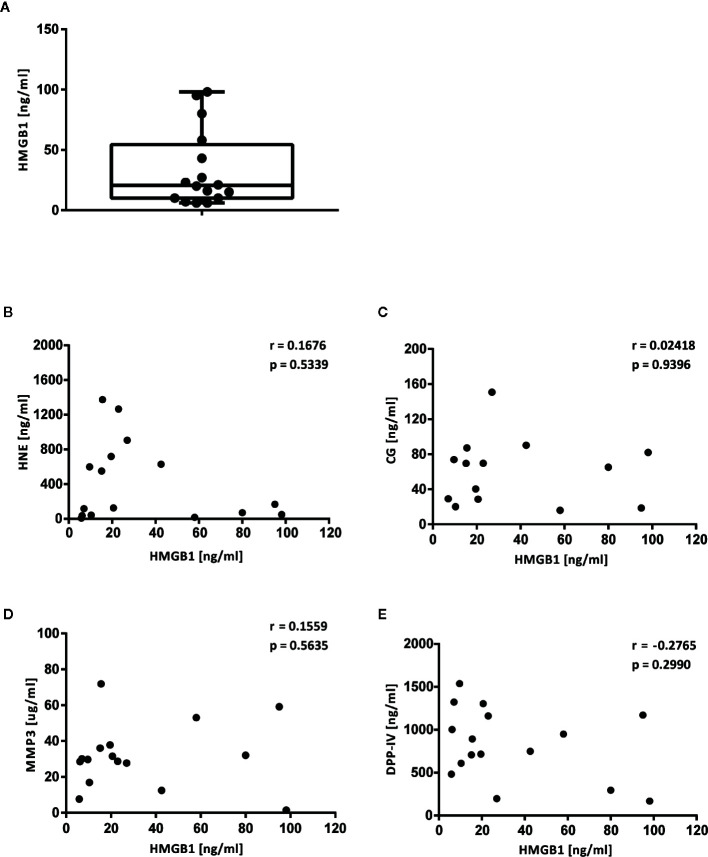
Levels of HMGB1, HNE, CG, MMP3 and DPP-IV in synovial fluid from JIA patients. Levels of HMGB1 and of the proteases investigated *in vitro* were defined in 16 synovial fluid samples from JIA patients. **(A)** Levels of HMGB1. **(B)** Levels of HNE. **(C)** Levels of CG. **(D)** Levels of MMP3. **(E)** Levels of DPP-IV. Direct correlations between HMGB1 levels and the level of each protease were assessed by Spearman’s correlation test.

We recorded the levels of HMGB1 in synovial fluid samples obtained from 16 JIA patients by ELISA in order to verify its presence in a biological fluid from an inflammatory condition. The levels ranged from 6 to 98 ng/ml; average 33.4 ng/ml, in agreement with previous reports (31, 32) (**Figure 6A**).

### HMGB1-Regulating Proteases Are Present in Synovial Fluid of JIA Patients

We confirmed the presence of the neutrophil-derived proteases, HNE and CG, in synovial fluid aliquots obtained from the 16 JIA patients in which we defined HMGB1 levels above (**Figures 6B, C**). HNE could be detected in all samples with levels ranging from 5.4 to 590.6 ng/ml with an average of 278.1 ng/ml. For CG, 2 samples had undetectable levels and the 14 positive samples had levels ranging from 18.7 to 150.8 pg/ml with an average of 60.2 pg/ml. The matrix metalloproteinase MMP3 was detected in all samples with levels ranging from 1.5 to 71.9 µg/ml with an average of 31.5 μg/ml (**Figure 6D**). DPP-IV could also be detected in all SF samples and ranged from 169 to 1536.9 ng/ml with an average of 828.9 ng/ml (**Figure 6E**).

We used the recorded average value for each analyzed protease and their molecular weight (HNE Mw, 28.5 kDa; CG Mw, 28.8 kDa; MMP3 Mw, 54.0 kDa; and DPP-IV Mw, 88.3 kDa) to compare the molar ratios of HMGB1 (Mw, 24.9 kDa) to proteases in synovial fluid. We could demonstrate that HNE, MMP-3, and DPP-IV are present in higher molar ratios in SF whereas the molar ratio for HMGB1:CG is lower in SF than the molar ratios used *in vitro* in this study (**Table 2**). Thus, from a stochiometric aspect, it is plausible that HMGB1 could be enzymatically processed by HNE and MMP-3 in arthritic joints. Considering the very rapid degradation of HMGB1 by CG *in vitro*, it is also plausible that the amount of CG present in SF, although lower that used in our *in vitro* experiments, is sufficient for processing of HMGB1.

**Table 2:** Molar ratios of proteases and HMGB1.

Protease	SF levels (ng/ml)	Mw (kDa)	Protease/HMGB1 ratio in SF	Protease/HMGB1 ratio *in vitro*
HNE	278.1	28.5	7.3:1	1:50
CG	0.06	28.8	0.002:1	1:80
MMP3	31500	54.0	435:1	1:22
DPP-IV	828.9	88.3	7:1	1:28
*HMGB1*	*33,4*	*24.9*		

Using the recorded average values for each investigated protease and for HMGB1 in synovial fluid samples from JIA patients, molar ratios of protease/HMGB1 were calculated.

### Levels of HMGB1 in Synovial Fluid Do Not Correlate With Levels of HNE, CG, or MMP-3

Correlation of HMGB1 levels with the levels of the respective proteases did not reveal a direct relationship between low levels of HMGB1 and presence of proteases (HNE vs HMGB1 r = −0.167, p = 0.534, CG vs HMGB1 r = 0.024, p = 0.940, MMP-3 vs HMGB1 r = 0.156, p = 0.564, DPP-IV vs HMGB1 r = −0.277, p = 0.299) (**Figures 6B–E**).

## Discussion

In this study we set out to test our hypothesis that the alarmin HMGB1 can be regulated by proteases associated with inflammatory conditions. Such proteolytic cleavage could either lead to the formation of fragments with altered, enhanced or antagonistic features or direct downregulation of HMGB1 activity through degradation. As a model inflammatory condition, we used chronic inflammatory arthritis. High levels of HMGB1 have been recorded in synovial fluid samples from both RA patients and JIA patients, which was also verified in this study.

Major cellular sources of proteases are neutrophils, the dominant cell type in synovial fluid, and activated synovial fibroblasts. We thus opted to study the ability of HNE, CG and MMP3, all derived from neutrophils or fibroblasts, to cleave HMGB1. Additionally, we investigated the HMGB1-cleaving properties of DPP-IV as this protease has been reported to cleave HMGB1 with implications for diabetes (26), and is detected at increased levels in arthritic synovium (33).

Our results demonstrate that three of the four studied proteases have the ability to cleave HMGB1. In our hands, DPP-IV, the investigated protease reported to cleave HMGB1 in a previous study, did not interact with HMGB1. This is in agreement with the protease prediction analysis we performed. Despite prolonged incubation time, full length HMGB1 incubated with DPP-IV was intact after 24 h using similar conditions as reported by Marchetti *et al*. These results are puzzling, and we do not have a good explanation. The only difference in experimental set up clear to us is the use of the commercially available full length HMGB1 from HMGBiotech used by Marchetti *et al*, and our in house produced full length HMGB1. Full length HMGB1 from HMGBiotech is tag free. Our in-house tag free HMGB1 has a GA scar in the N-terminus. Whether this affects the DDP-IV activity is presently unclear.

Conversely, CG induced a rapid and total fragmentation of HMGB1. Multiple smaller peptides were already evident after 5 min incubation. This result was somewhat expected, as CG cleaves substrates with Glu, Lys, Trp, and Phe in position P1 and with no selectivity of any amino acid in position P1′ (45,50). HMGB1 contains 35 Glu residues, 43 Lys residues, 2 Trp residues and 9 Phe residues. Our results implicate that CG might be a rapid and efficient mediator of HMGB1 removal through degradation during inflammatory conditions.

Digestion of HMGB1 with both HNE and MMP3 resulted in the generation of larger HMGB1 fragments. Interestingly, Both HNE and MMP3 generated fragments lacking the C-terminal tail. Earlier studies have demonstrated that the C-terminal tail interacts with both box regions and the linker region of HMGB1 in a dynamic fashion, resulting in tail-bound and tail-unbound conformations (10, 11). Binding of the tail to the boxes modulates the interaction between HMGB1 and DNA and also regulates acetylation of HMGB1. As the receptor-binding domains for RAGE and TLR4 in HMGB1 are located within either the boxes or in the C-terminal linker region (see **Figure 1**), an HMGB1 fragment lacking its C-tail could have altered receptor associations. In support of this are recent findings that only HMGB1 lacking its C-terminal tail binds to TLR2 and to TLR5 (21, 34). Similarly, removal of the N-terminal region of the alarmin IL-33 by HNE or by CG increases the ligand-binding activity of the resulting fragment (35).

**Figure 7:**
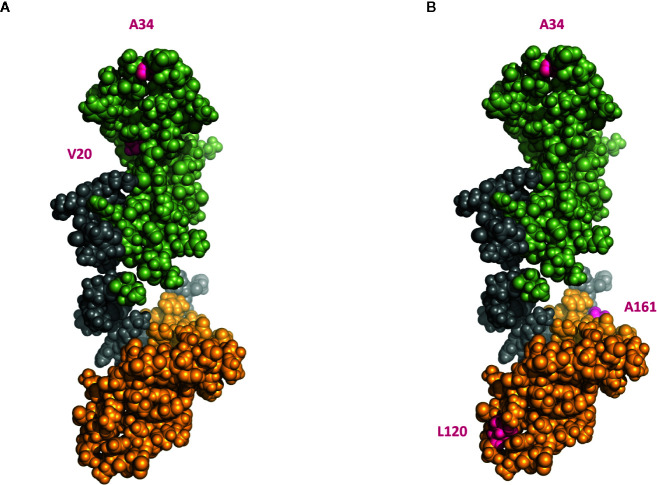
A 3D model indicating proposed cleavage sites at HMGB1 by HNE and MMP3. **(A)** 3D model of HMGB1 showing predicted HNE cleavage sites at positions V20 and A34 **(B)** 3D model of HMGB1 showing predicted MMP3 cleavage sites at positions A34, L120 and A161 (pink). Box A is marked in green, box B marked in yellow. The model is based on solution structure of the tandem HMG box domain from Human High mobility group protein B1 aa 1–166, #2YRQ in the RCSB Protein Data Bank.

Both HNE and MMP3 digestion of HMGB1 resulted in cleavage at A34, creating a fragment lacking the N-terminal part of the molecule in addition to lacking the C-terminal tail (**Figure 7**). This could alter the accessibility of box A for receptor interactions. This is of interest as recombinantly produced “free” box A acts as an antagonist to HMGB1 in multiple models of inflammation. To define the exact P1/P1′ positions, point mutated HMGB1 needs to be produced and subjected to cleavage. This was however outside the scope for this study.

It is notable in the Western blotting experiments that incubation of HNE with HMGB1 results in the formation of a large Mw complex (**Figure 2B**, time points 10 and 20 min). The Mw corresponds to a dimer of HNE and HMGB1, most likely formed by covalent bonds as the presence of reducing agents in the sample buffer did not dissolve the complex. It is a known feature of endopeptidases to form covalent complexes with substrates during the catalytic reaction. The covalent bond is subsequently broken and the enzyme regenerated (36). In our study the high molecular weight complex was absent from the 60-min time point.

Our Western blotting analyses were somewhat restricted by the lack of a commercially available box B-specific antibody and neither have we managed to produce such an antibody in-house despite several attempts. Additionally, it has to be noted that we have worked with a recombinant HMGB1 most likely being oxidized as it was produced with a histidine tag, without any reducing agent in the buffers and neither did it induce cytokine production when tested (data not shown).

In order to assess the biological relevance of our *in vitro* findings we first verified the presence of the investigated proteases during an inflammatory condition where HMGB1 is regarded as a pathogenic mediator; arthritis. We could demonstrate that all four investigated proteases were present in JIA synovial fluid. This is the first report of HNE, CG, MMP3 and DPP-IV levels in JIA synovial fluid as well as their co-existence with HMGB1 in a biological fluid.

Based on our findings that HMGB1 is a substrate for 3 proteases readily detected in arthritic joints it might be surprising that HMGB1 itself can be detected in arthritic synovial fluid. A technical reason could be that the antibody pair in the commercial ELISA assay used recognizes not only full length HMGB1 but also fragments of the protein. A more likely reason, however, is the intricate system of protease-binding and inhibiting proteins present in arthritic joints.

In conclusion, we report the novel finding that HMGB1 can be regulated by proteases associated with inflammation and arthritis. Using literature searches, protease specificity prediction servers and by performing *in vitro* studies we report that CG rapidly degrades HMGB1 while HNE and MMP3 processing had a slower kinetics resulting in larger fragments. We propose that HNE primarily cleaves HMGB1 at A170 and A34 in position P1, and that MMP3 primarily cleaves HMGB1 in position A161. Both HNE and MMP3 processing hence results in cleavage in the linker region, removing the C-terminal tail. Such truncation of HMGB1 has previously been reported by us and others to affect HMGB1-receptor interactions. Further processing resulted in fragments lacking the N-terminal region, a feature described for other inflammatory mediators to regulate their activity.

For the first time, we report the presence of HNE, CG, MMP3 and DPP-IV in synovial fluid obtained from JIA patients. Although the levels of each protease did not correlate with the levels of HMGB1, our study suggests that proteolytic cleavage of HMGB1 can be a downregulatory mechanism of HMGB1 activity during arthritis. Future studies are needed to clarify functional consequences of the observed fragments produced by the investigated enzymes.

## Data Availability Statement

The data sets generated for this study are available on request to the corresponding author.

## Ethics Statement

This study was carried out in accordance with the recommendations of North Ethical Committee in Stockholm, Sweden with written informed consent from all subjects. All subjects gave written informed consent in accordance with the Declaration of Helsinki. The protocol was approved by North Ethical Committee in Stockholm, Sweden.

## Author Contributions

AS, PL, and HH conceived and designed the study. AS, MR, MN, and LK performed the experiments. AS, PL, and HH wrote the paper. MR, MN, and LK reviewed and edited the manuscript. All authors contributed to the article and approved the submitted version.

## Funding

This study was supported by grants to HEH by the Swedish Research Council (2015-02776-3 and 2018-028885), the Swedish Cancer Society, the Swedish Rheumatism Association and the Stockholm County Council.

## Conflict of Interest

The authors declare that the research was conducted in the absence of any commercial or financial relationships that could be construed as a potential conflict of interest.

## References

[1] Andersson U Tracey KJ HMGB1 is a therapeutic target for sterile inflammation and infection. Annu Rev Immunol (2011) 29:139–62. 10.1146/annurev-immunol-030409-101323 PMC4536551 21219181

[2] Andersson U Yang H Harris H High-mobility group box 1 protein (HMGB1) operates as an alarmin outside as well as inside cells. Semin Immunol (2018) 38:40–8. 10.1016/j.smim.2018.02.011 29530410

[3] Pullerits R Jonsson IM Verdrengh M Bokarewa M Andersson U Erlandsson-Harris H High mobility group box chromosomal protein 1, a DNA binding cytokine, induces arthritis. Arthritis Rheum (2003) 48(6):1693–700. 10.1002/art.11028 12794838

[4] Kokkola R Li J Sundberg E Aveberger AC Palmblad K Yang H Successful treatment of collagen-induced arthritis in mice and rats by targeting extracellular high mobility group box chromosomal protein 1 activity. Arthritis Rheum (2003) 48(7):2052–8. 10.1002/art.11161 12847700

[5] Ostberg T Kawane K Nagata S Yang H Chavan S Klevenvall L Protective targeting of high mobility group box chromosomal protein 1 in a spontaneous arthritis model. Arthritis Rheum (2010) 62(10):2963–72. 10.1002/art.27590 20533288

[6] Schierbeck H Lundback P Palmblad K Klevenvall L Erlandsson-Harris H Andersson U Monoclonal anti-HMGB1 (high mobility group box chromosomal protein 1) antibody protection in two experimental arthritis models. Mol Med (2011) 17(9-10):1039–44. 10.2119/molmed.2010.00264 PMC3188862 21666956

[7] Andersson U Yang H Harris H Extracellular HMGB1 as a therapeutic target in inflammatory diseases. Expert Opin Ther Targ (2018) 22(3):263–77. 10.1080/14728222.2018.1439924 29447008

[8] Venereau E Casalgrandi M Schiraldi M Antoine DJ Cattaneo A De Marchis F Mutually exclusive redox forms of HMGB1 promote cell recruitment or proinflammatory cytokine release. J Exp Med (2012) 209(9):1519–28. 10.1084/jem.20120189 PMC3428943 22869893

[9] Blair RH Horn AE Pazhani Y Grado L Goodrich JA Kugel JF The HMGB1 C-Terminal Tail Regulates DNA Bending. J Mol Biol (2016) 428(20):4060–72. 10.1016/j.jmb.2016.08.018 PMC5642108 27558111

[10] Knapp S Muller S Digilio G Bonaldi T Bianchi ME Musco G The long acidic tail of high mobility group box 1 (HMGB1) protein forms an extended and flexible structure that interacts with specific residues within and between the HMG boxes. Biochemistry (2004) 43(38):11992–7. 10.1021/bi049364k 15379539

[11] Stott K Watson M Howe FS Grossmann JG Thomas JO Tail-mediated collapse of HMGB1 is dynamic and occurs via differential binding of the acidic tail to the A and B domains. J Mol Biol (2010) 403(5):706–22. 10.1016/j.jmb.2010.07.045 20691192

[12] Yang H Hreggvidsdottir HS Palmblad K Wang H Ochani M Li J A critical cysteine is required for HMGB1 binding to Toll-like receptor 4 and activation of macrophage cytokine release. Proc Natl Acad Sci U S A (2010) 107(26):11942–7. 10.1073/pnas.1003893107 PMC2900689 20547845

[13] Yang H Ochani M Li J Qiang X Tanovic M Harris HE Reversing established sepsis with antagonists of endogenous high-mobility group box 1. Proc Natl Acad Sci U S A (2004) 101(1):296–301. 10.1073/pnas.2434651100 14695889 PMC314179

[14] Yang H Liu H Zeng Q Imperato GH Addorisio ME Li J Inhibition of HMGB1/RAGE-mediated endocytosis by HMGB1 antagonist box A, anti-HMGB1 antibodies, and cholinergic agonists suppresses inflammation. Mol Med (2019) 25:13. 10.1186/s10020-019-0081-6 30975096 PMC6460792

[15] Zetterstrom CK Bergman T Rynnel-Dagoo B Erlandsson Harris H Soder O Andersson U High mobility group box chromosomal protein 1 (HMGB1) is an antibacterial factor produced by the human adenoid. Pediatr Res (2002) 52(2):148–54. 10.1203/00006450-200208000-00004 12149489

[16] Hreggvidsdottir HS Ostberg T Wahamaa H Schierbeck H Aveberger AC Klevenvall L The alarmin HMGB1 acts in synergy with endogenous and exogenous danger signals to promote inflammation. J Leukocyte Biol (2009) 86(3):655–62. 10.1189/jlb.0908548 19564572

[17] Yang H Wang H Wang Y Addorisio M Li J Postiglione MJ The haptoglobin beta subunit sequesters HMGB1 toxicity in sterile and infectious inflammation. J Internal Med (2017) 282(1):76–93. 10.1111/joim.12619 28464519 PMC5477782

[18] Abeyama K Stern DM Ito Y Kawahara K Yoshimoto Y Tanaka M The N-terminal domain of thrombomodulin sequesters high-mobility group-B1 protein, a novel antiinflammatory mechanism. J Clin Invest (2005) 115(5):1267–74. 10.1172/JCI22782 PMC1077171 15841214

[19] Hagiwara S Iwasaka H Matsumoto S Hasegawa A Yasuda N Noguchi T In vivo and in vitro effects of the anticoagulant, thrombomodulin, on the inflammatory response in rodent models. Shock (2010) 33(3):282–8. 10.1097/SHK.0b013e3181b0ef7b 19536047

[20] Van de Wouwer M Plaisance S De Vriese A Waelkens E Collen D Persson J The lectin-like domain of thrombomodulin interferes with complement activation and protects against arthritis. J Thromb Haemostasis JTH (2006) 4(8):1813–24. 10.1111/j.1538-7836.2006.02033.x 16879225

[21] Aucott H Sowinska A Harris HE Lundback P Ligation of free HMGB1 to TLR2 in the absence of ligand is negatively regulated by the C-terminal tail domain. Mol Med (2018) 24(1):19. 10.1186/s10020-018-0021-x 30134807 PMC6016865

[22] LeBlanc PM Doggett TA Choi J Hancock MA Durocher Y Frank F An immunogenic peptide in the A-box of HMGB1 protein reverses apoptosis-induced tolerance through RAGE receptor. J Biol Chem (2014) 289(11):7777–86. 10.1074/jbc.M113.541474 PMC3953289 24474694

[23] Song J Tan H Perry AJ Akutsu T Webb GI Whisstock JC PROSPER: an integrated feature-based tool for predicting protease substrate cleavage sites. PLoS One (2012) 7(11):e50300. 10.1371/journal.pone.0050300 23209700 PMC3510211

[24] Qin S Wang H Yuan R Li H Ochani M Ochani K Role of HMGB1 in apoptosis-mediated sepsis lethality. J Exp Med (2006) 203(7):1637–42. 10.1084/jem.20052203 PMC2118346 16818669

[25] Liu K Mori S Takahashi HK Tomono Y Wake H Kanke T Anti-high mobility group box 1 monoclonal antibody ameliorates brain infarction induced by transient ischemia in rats. FASEB J (2007) 21(14):3904–16. 10.1096/fj.07-8770com 17628015

[26] Marchetti C Di Carlo A Facchiano F Senatore C De Cristofaro R Luzi A High mobility group box 1 is a novel substrate of dipeptidyl peptidase-IV. Diabetologia (2012) 55(1):236–44. 10.1007/s00125-011-2213-6 21656024

[27] Schilling O Overall CM Proteome-derived, database-searchable peptide libraries for identifying protease cleavage sites. Nat Biotechnol (2008) 26(6):685–94. 10.1038/nbt1408 18500335

[28] Turk BE Huang LL Piro ET Cantley LC Determination of protease cleavage site motifs using mixture-based oriented peptide libraries. Nat Biotechnol (2001) 19(7):661–7. 10.1038/90273 11433279

[29] Korkmaz B Horwitz MS Jenne DE Gauthier F Neutrophil elastase, proteinase 3, and cathepsin G as therapeutic targets in human diseases. Pharmacol Rev (2010) 62(4):726–59. 10.1124/pr.110.002733 PMC2993259 21079042

[30] Fu Z Thorpe M Akula S Chahal G Hellman LT Extended Cleavage Specificity of Human Neutrophil Elastase, Human Proteinase 3, and Their Distant Ortholog Clawed Frog PR3-Three Elastases With Similar Primary but Different Extended Specificities and Stability. Front Immunol (2018) 9:2387. 10.3389/fimmu.2018.02387 30459762 PMC6232827

[31] Schierbeck H Pullerits R Pruunsild C Fischer M Holzinger D Laestadius A HMGB1 levels are increased in patients with juvenile idiopathic arthritis, correlate with early onset of disease, and are independent of disease duration. J Rheumatol (2013) 40(9):1604–13. 10.3899/jrheum.120987 23858044

[32] Pullerits R Schierbeck H Uibo K Liivamagi H Tarraste S Talvik T High mobility group box protein 1-A prognostic marker for structural joint damage in 10-year follow-up of patients with juvenile idiopathic arthritis. Semin Arthritis Rheum (2017) 46(4):444–50. 10.1016/j.semarthrit.2016.08.017 27756498

[33] Gotoh H Hagihara M Nagatsu T Iwata H Miura T Activities of dipeptidyl peptidase II and dipeptidyl peptidase IV in synovial fluid from patients with rheumatoid arthritis and osteoarthritis. Clin Chem (1989) 35(6):1016–8. 10.1093/clinchem/35.6.1016 2567214

[34] Das N Dewan V Grace PM Gunn RJ Tamura R Tzarum N HMGB1 Activates Proinflammatory Signaling via TLR5 Leading to Allodynia. Cell Rep (2016) 17(4):1128–40. 10.1016/j.celrep.2016.09.076 PMC5087801 27760316

[35] Lefrancais E Roga S Gautier V Gonzalez-de-Peredo A Monsarrat B Girard JP IL-33 is processed into mature bioactive forms by neutrophil elastase and cathepsin G. Proc Natl Acad Sci U S A (2012) 109(5):1673–8. 10.1073/pnas.1115884109 PMC3277172 22307629

[36] Dexpert J Delain E Piriou B Pochon F The covalent and non-covalent binding modes of elastase with alpha 2-macroglobulin influence the conformation of the protease. FEBS Lett (1987) 225(1-2):223–7. 10.1016/0014-5793(87)81162-6 2446921

