# Supplementary figures and images for: Cleavage of HMGB1 by Proteolytic Enzymes Associated with Inflammatory Conditions
Source: Front Immunol. 2020 Dec 16;11:448262. doi: 10.3389/fimmu.2020.448262 (PMC7772184; doi:10.3389/fimmu.2020.448262)

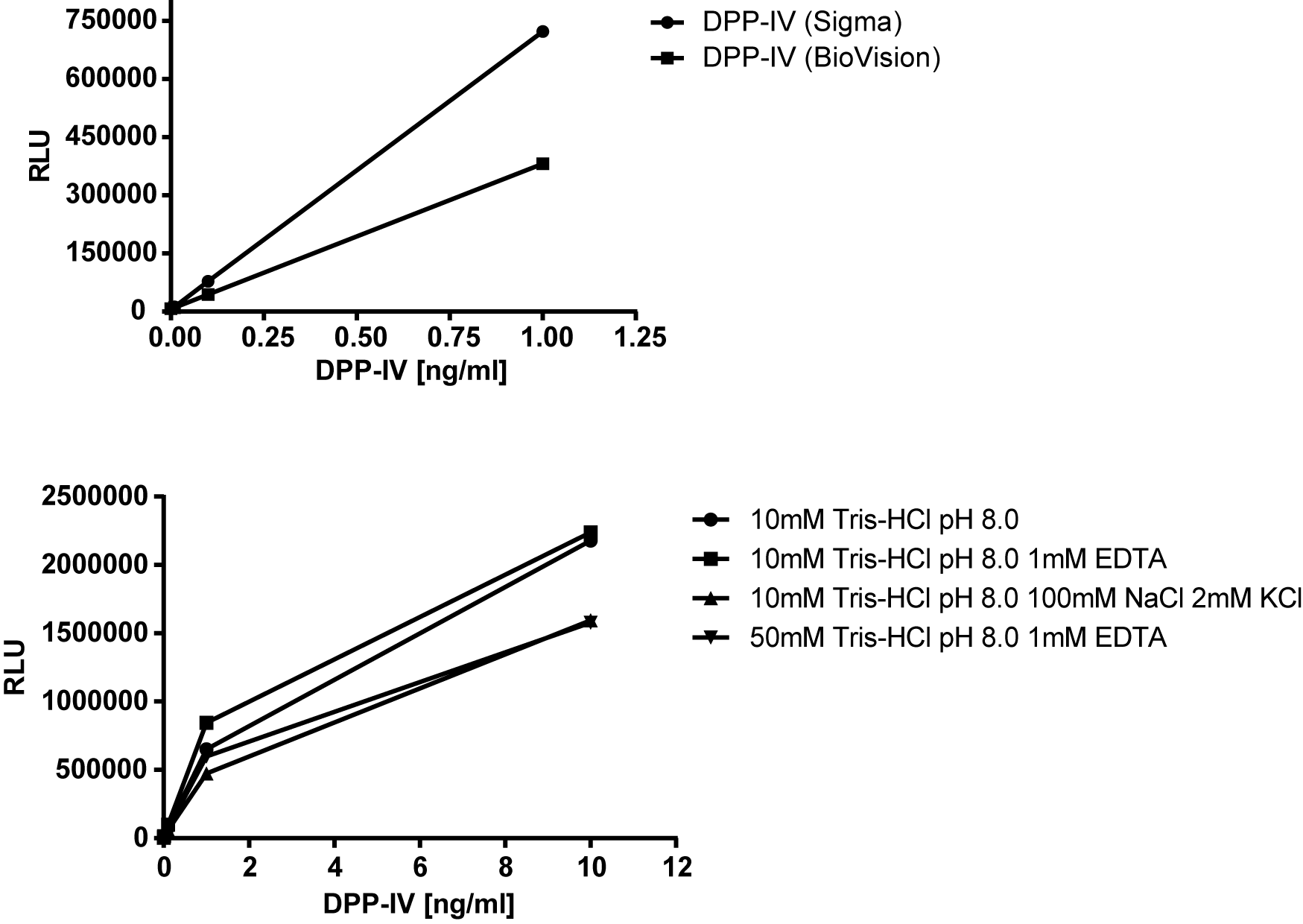

Supplement: Supplementary file 1 [file Image_1.tif]
